# Differentiated Thyroid Cancer: Management of Patients with Radioiodine Nonresponsive Disease

**DOI:** 10.1155/2012/618985

**Published:** 2012-02-28

**Authors:** Naifa Lamki Busaidy, Maria E. Cabanillas

**Affiliations:** Department of Endocrine Neoplasia and Hormonal Disorders, University of Texas MD Anderson Cancer Center, 1515 Holcombe Boulevard, Unit 1461, Houston, TX 77030, USA

## Abstract

Differentiated thyroid carcinoma (papillary and follicular) has a favorable prognosis with an 85% 10-year survival. The patients that recur often require surgery and further radioactive iodine to render them disease-free. Five percent of thyroid cancer patients, however, will eventually succumb to their disease. Metastatic thyroid cancer is treated with radioactive iodine if the metastases are radioiodine avid. Cytotoxic chemotherapies for advanced or metastatic noniodine avid thyroid cancers show no prolonged responses and in general have fallen out of favor. Novel targeted therapies have recently been discovered that have given rise to clinical trials for thyroid cancer. Newer aberrations in molecular pathways and oncogenic mutations in thyroid cancer together with the role of angiogenesis in tumor growth have been central to these discoveries. This paper will focus on the management and treatment of metastatic differentiated thyroid cancers that do not take up radioactive iodine.

## 1. Introduction

Thyroid carcinoma is the most common endocrine malignancy with a prevalence of 335,000 and incidence of 37,200 in the United States in 2009 [1]. Differentiated thyroid carcinoma, namely papillary and follicular thyroid carcinoma, makes up about 94% of these cases. Despite the generally good prognosis of thyroid carcinoma, about 5% of patients will develop metastatic disease which fails to respond to radioactive iodine, exhibiting a more aggressive behavior. These patients will die of their disease [1–4]. 

85% of patients with differentiated thyroid carcinomas are cured with surgery, radioactive iodine, and TSH suppression. Of those that recur, the vast majority will recur in the neck, and best treatment options are surgical with potential further radioactive iodine. A small percentage of patients will develop or present with metastases and are more difficult to treat. When metastases have radioiodine avidity, prognosis is better, and further radioactive iodine may be used. However, when multiple doses of radioactive iodine have been tried or the patient has nonradioactive iodine avid disease, other options need to be considered. This paper will aim to discuss the treatment options of those patients with nonradioiodine avid, recurrent, or metastatic differentiated thyroid cancer.

## 2. Diagnosis of Recurrent/Metastatic Disease Extent

Screening ultrasound of the neck and tumor marker (thyroglobulin) should be performed on all patients with differentiated thyroid cancer,per accepted guidelines [5]. The finding of an elevated thyroglobulin or thyroglobulin antibody in the face of a negative radioactive iodine scan is indicative of non-radioiodine avid residual or recurrent disease. Both ultrasound of the neck and thin spiral CT of the chest should be performed for detection of disease. If symptoms occur or the thyroglobulin is out of proportion to the amount of disease seen, other imaging can be ordered as dictated by the clinical scenario. Other imaging modalities include MRI of the brain, spine, bone scan, and ^18^FDG-PET/CT scans.  Table 1 summarizes the imaging modalities used in thyroid cancer surveillance. 

### 2.1. Role of Ultrasound

Ultrasonography (U/S) of the neck (thyroid bed and cervical neck compartments), as opposed to RAI scans, is recommended in the followup of these patients. This shift in practice is due to the fact that many recurrent tumors lose the ability to capture iodine, leading to false negatives. As many as half of the patients with findings of recurrence on U/S may have no uptake on radioiodine scanning or may have an undetectable serum thyroglobulin [6]. 

Recurrence of papillary thyroid carcinoma is most commonly in the neck (thyroid bed and lymph nodes), and hence, ultrasound is the mainstay of routine followup of these patients. U/S can be used to accurately diagnose and identify lesions in the neck as small as 3 mm. Routine use of U/S in the 3- to 12-month monitoring of patients with extrathyroidal invasion or local-regional nodal metastases [7, 8] is now recommended as part of consensus guidelines [5]. Although U/S can aid in distinguishing benign lesions from malignant lesions, FNA (U/S guided) is most helpful to definitively prove recurrent cancer. Thyroglobulin can be measured in the washout of the needle taken from neck lymph nodes [9–11]. This is especially helpful in cases where the FNA specimen is nondiagnostic. 

### 2.2. CT and MRI

Other imaging techniques that can be used in individual cases of thyroid cancer followup include CT scan of the neck with IV contrast, CT scan of the chest, and magnetic resonance imaging (MRI). MRI and CT scan of the neck play important roles in the detection of recurrent disease although the sensitivity of these is not as well established as ultrasound for the detection of true thyroid cancer recurrences in the neck. CT and MRI of the neck are not recommended for routine use in the detection of recurrent disease but have the advantage of being much less operator dependent. If good ultrasonography is not readily available or deep posterior neck disease is suspected, CT and MRI of the neck can be used for the detection of disease. 

The most common place for papillary thyroid carcinoma to metastasize outside the neck is the chest. CT scan of the chest may show macro- and micronodular pulmonary metastases that do not routinely take up iodine. This cross-sectional imaging of the chest is used for long-term followup when lung metastases are known or suspected based on elevations in thyroglobulin. 

CT and MRI scans of other less commonly found sites of distant metastases include imaging of the brain, spine, abdomen, and pelvis as the clinical scenario dictates based on symptoms, clinical suspicion, or prior to initiation of various therapies.

### 2.3. ^18^FDG PET-CT

Fluorodeoxyglucose positron-emission tomography (FDG-PET or PET)/CT imaging is an increasingly more useful tool in the detection of radioiodine-negative, thyroglobulin-positive thyroid cancer [12–15]. Thyroid carcinomas with little to no iodine activity tend to have higher glucose metabolism and positive FDG-PET scans [12, 16, 17]. This tends to be representative of tumor dedifferentiation. Patients with larger volumes of FDG-avid disease or higher SUVs are less likely to respond to radioiodine and have a higher mortality over a 3-year followup compared with the patients with no FDG uptake [18, 19]. Tumors that take up radioactive iodine are less likely to yield positive FDG PET scans [20]. 

PET/CT scans can be used for detection of occult recurrences or metastases [16, 21, 22] or to provide information about the biology of the metastatic disease and prognostic information. The latter is not standard practice, but several studies have now shown that FDG-PET correlates with the overall survival [14, 15, 21]. This information may be helpful to decide which patients warrant systemic treatment for their metastatic disease if they are refractory/resistant to radioactive iodine or have reached the maximum benefit from this treatment. 

^18^FDG-PET has been approved for reimbursement for the detection of occult thyroid cancer in patients who have a thyroglobulin greater than 10 ng/mL and have negative radioiodine imaging. ^18^FDG-PET CTs are also used in those patients whose cancers are very poorly differentiated and make no thyroglobulin.

The overall sensitivity, specificity, and accuracy of ^18^F-FDG PET/CT in one series of 59 patients with radioiodine-negative, thyroglobulin-positive, recurrent disease were 68.4%, 82.4%, and 73.8%, respectively [23]. Other studies have shown a sensitivity of 70–95% and a specificity of 77–100% [12, 13]. FDG-PET is not sensitive enough to detect subcentimeter metastases, as it is common in metastatic papillary thyroid carcinoma and should be used in conjunction with CT chest imaging. 

TSH stimulates ^18^FDG uptake by differentiated thyroid carcinoma [24], suggesting that PET scans may be more sensitive after TSH stimulation with rhTSH or withdrawal of thyroid hormone [24–26]. While rhTSH-stimulated PET-CT identified more total FDG-avid lesions compared to nonstimulated FDG-PET CT in a large multicenter study, it changed treatment planning only 6% of the time [27]. 

False positives, such as infections or granulomatous diseases/sarcoid or postoperative changes due to inflammation, amongst others, have been reported in thyroid cancer about 11–25% of the time suggesting that the malignant nature of the disease should be confirmed prior to further therapy [27–30]. 

## 3. Treatment of Advanced or Metastatic Thyroid Cancer

Most patients with differentiated thyroid cancers are rendered free of disease after surgery, radioactive iodine, and thyroid hormone suppression. Approximately 15–20% of patients will recur locoregionally or have distant metastases. Although it is the most effective medical treatment for differentiated thyroid carcinoma, only about 50–80% of primary tumors and their metastases take up radioactive iodine [7, 31–34], rendering this therapy ineffective in most cases. Thus, other treatment modalities such as surgery, external beam radiation, percutaneous ethanol injection therapy (PEIT), and systemic chemotherapy are indicated.  Table 2 summarizes the indications for each of these therapies.

### 3.1. Surgery

Surgery in advanced thyroid carcinomas is most commonly used for recurrent neck metastases and metastasectomies in selected sites. Recurrences in the neck are most commonly seen in the thyroid bed or regional lymph nodes. Although most occur within the first five years after diagnosis, late recurrences do occur. In one study with 40-year followup, 35% of patients recurred. Two-thirds of them were within the first decade after initial therapy, and two thirds were locoregional [35].

Surgery is considered first-line therapy in patients with gross nodal or recurrent neck disease. This can be followed by further radioactive iodine (if the recurrent tumors took up radioiodine prior to surgery) and thyroid hormone suppression. One-third to one half of patients may be free of disease in short-term followup [36]. If the gross tumors do not take up radioactive iodine from previous posttreatment scans or preoperative radioiodine scans are negative, further postoperative radioactive iodine will be of limited benefit and may increase the side effects of further iodine. Adverse events from further radioactive iodine include xerostomia, nasolacrimal duct obstruction, and secondary malignancies [37–42].

Surgery alone with complete ipsilateral compartmental dissections of involved areas or modified neck dissections as opposed to “berry picking” or selective lymph node resection procedures or ethanol ablation may be of benefit [43, 44]. It is not evident that recurrent locoregional disease in the setting of distant metastatic disease should be resected, unless there is airway or an other vital structural compromise. If the tumors invade the upper aerodigestive tract, a combined treatment modality of surgery and ^131^I (if tumors take up radioactive iodine) and/or adjuvant external beam radiotherapy is advised [45–48]. Due to potential morbidity from surgical resection of recurrent disease, these patients should be referred to centers with expertise in this area.

Surgery is also considered for isolated metastases, metastases in bone (especially if long bones, spine, or weight bearing), and brain (see Section 3.3 below).

### 3.2. External Beam Radiotherapy

External beam radiotherapy (EBRT) has a very specific role in the treatment of papillary thyroid carcinoma. Although it is somewhat controversial, retrospective studies have shown that it may be an effective adjuvant therapy to prevent local-regional recurrence in patients 45 years of age and older with locally invasive papillary carcinoma after surgery [49, 50]. Ten-year local relapse-free rates (93% versus 78%) and disease-specific survival rates (100% versus 95%) were higher in a subgroup of patients with papillary histology and presumed microscopic disease treated with EBRT [49]. Doses in the range of 40–50 Gy may aid in local-regional control in patients with papillary thyroid carcinoma who are over 45 years of age and have incomplete resection near the aerodigestive tract and/or those with gross extrathyroidal invasion with presumed microscopic residual disease. EBRT is generally avoided in patients under 45 years of age both because of their good prognosis and the potential late side effects of therapy including secondary malignancies. External beam radiation may also preclude further surgery in the future if the tumor recurs. 

Acute complications of external beam radiotherapy include esophagitis and tracheitis. Long-term complications include neck fibrosis, xerostomia, dental decay, osteoradionecrosis, and the risk of tracheal stenosis [34]. Newer techniques to deliver radiation with fewer adverse events are being used in the treatment of cancer, including intensity-modulated radiation therapy (IMRT). Limited data in thyroid cancer with short-term followup suggests similar outcomes and may reduce chronic morbidity relative to conventional EBRT [51]. Many centers currently use IMRT as the radiation treatment of choice for thyroid carcinomas requiring EBRT [52].

### 3.3. Metastatic Sites Requiring Special Attention

Although most patients with metastatic disease will need systemic therapy, metastatic disease to certain sites deserves special attention.

#### 3.3.1. CNS Metastases

Brain metastases more often occur in elderly individuals with more advanced disease and have an overall poorer prognosis [53]. Surgical resection significantly improves median overall survival from four to 22 months in patients with 1 or more brain metastases [53]. Current guidelines recommend resection when one CNS lesion is present [54]. Radioiodine therapy and/or external beam radiotherapy (with steroids to minimize tumor swelling) should be considered after surgical resection [55]. If CNS lesions are not surgically resectable or the morbidity from surgery is unacceptable, whole brain radiotherapy for numerous lesions or gamma knife radiosurgery to selected lesions should be used in conjunction with radioiodine if the tumor concentrates iodine [56]. If radioiodine is to be used, prior radiotherapy and concomitant steroids should be strongly considered to decrease tumor swelling [57].

#### 3.3.2. Bone Metastases

Although bone lesions tend to concentrate radioiodine as well as lungs, there is complete resolution in less than 10% of the time. Metastases that cause pain or compression of spinal cord or other vital organs necessitate treatment. Symptoms from painful bone lesions or spinal-cord-compressing lesions may be relieved by surgical treatment. External beam radiation therapy (EBRT) or gamma knife radiosurgery has also been used successfully to render bone lesions pain-free. Arterial embolization has been used with successful reduction in pain and neurologic symptoms and can be used in conjunction with external beam radiation [58]. ^131^I treatment may follow surgical resection of distant metastatic disease if the tumor takes up radioactive iodine. A recent study found that patients with solitary bony metastases treated with I131 and surgery had a better prognosis than those who did not [59].

Intravenous bisphosphonates (pamidronate or zoledronic acid) are prescribed for painful bony metastases with some success as well. Orita et al., retrospectively, examined 50 patients with bony metastases from DTC and found that those who had received monthly infusions of zoledronic acid had significantly fewer-skeletal related events (defined as fracture, spinal cord compression, and hypercalcemia) than those who did not receive this drug [60]. Whether this treatment slows progression of bone metastases is not known. The most common adverse event associated with intravenous bisphosphonates is a transient flu-like syndrome, usually associated with the first administration, with symptoms dissipating and disappearing with subsequent infusions. Osteonecrosis of the jaw is less common but serious adverse event associated with intravenous bisphosphonates. 

### 3.4. Systemic Therapy

Lack of RAI uptake by distant metastases confers a poor prognosis. For example, patients with no RAI uptake in the lungs have a 10-year survival rate of 25% compared with 76% in those whose lung metastases have RAI uptake [61]. Pulmonary metastases that do not take up radioactive iodine do not typically respond to that radionuclide therapy, and these patients are at high risk of death [62]. 

Most diagnostic scan-negative, thyroglobulin-positive patients who have disease seen on other imaging modalities are not rendered disease-free by repeat radioiodine treatments, although tumor burden may decrease [63]. No survival advantage nor decrease in morbidity has been seen with repeat radioactive iodine therapies. Repeated doses of radioactive iodine have been used in patients considered to have radioactive noniodine-avid, thyroglobulin-positive disease with little clinical benefit [64]. Although controversial, a single dose of 100–150 mCi of radioactive iodine therapy can be given to a patient with elevated thyroglobulin and negative diagnostic scan. A posttreatment scan should be done, and if negative, further radioactive iodine should be avoided.

Repeated radioiodine therapy has adverse events including xerostomia, nasolacrimal duct obstruction with epiphora, and secondary malignancies [37–42]. Further radioactive iodine therapy should generally be avoided in these patients, and the use of systemic agents should be considered [65]. 

Because metastatic differentiated thyroid cancer can be stable and quiescent for many years, only patients with progressive or symptomatic disease should be treated with other systemic treatments. Systemic therapy with targeted agents or cytotoxic chemotherapy is the usual treatment of choice for RAI-refractory, progressive distant metastatic disease. 

Clinical trials should consider first-line therapy for those patients who do not take up radioactive iodine. If a clinical trial is not available or the patient is not suitable for one, then off-label use of targeted therapies such as pazopanib, sorafenib, sunitinib, or cytotoxic chemotherapy should be considered [54].

#### 3.4.1. Cytotoxic Chemotherapy

Traditional cytotoxic chemotherapies such as doxorubicin, taxol, and cisplatin are associated with a 25–37% partial response rate with rare complete remission [66–69]. Due to toxic side effects, short duration of responses, and low response rates, systemic cytotoxic chemotherapy is reserved for patients with rapidly progressive metastatic disease that is not suitable or nonresponsive to surgery, radioiodine, and external beam radiotherapy and those who cannot enter into clinical trials or use targeted agents (see below). 

Systemic chemotherapy is used in certain cases of widespread progressive disease that is radioiodine resistant although available regimens have not been well studied and are not very effective to date. Doxorubicin is associated with a response rate of up to 40% for progressive differentiated cancers that do not respond to radioactive iodine [70, 71]. The recommended dosage is 60–75 mg/m^2^ every 3 weeks. Combination therapies are also used, but data are limited because of the small number of patients in reported series. Doxorubicin, epirubicin, taxol, and cisplatin have all been used in various combinations; responses do not seem to be any better than single agent with increased toxicities [68, 69, 72]. Response rates vary from 25 to 37% with mostly partial responses. Doxorubicin has also been used as a radiation sensitizer with not much different results from radiation alone. Patients with advanced progressive radioiodine nonresponsive disease should be considered for participation in clinical trials.

#### 3.4.2. Newer Targeted Therapies

Newer approaches to thyroid carcinoma therapy include inhibition of the various metabolic pathways found to be altered in these cancerous cells. Prior to the discussion of the available and tried targeted therapies below, we briefly summarize the known aberrant pathways.

**(1) Mutations and Promotion of Tumor Growth**

Follicular cell tumorigenesis pathways have been key to the development of clinical trials testing novel therapies in the treatment of thyroid cancer. Mutations in either BRAF, RAS or RET/PTC rearrangements are present in most differentiated thyroid cancers [73]. Chromosomal rearrangement of the gene encoding the transmembrane tyrosine kinase receptors *ret* and *trk* is one identified early step in the development of these tumors. RET/PTC genetic alterations have been found in 40% and 60% of papillary carcinomas in adults and children, respectively, and are the most common mutation found in the Chernobyl radiation-induced thyroid carcinomas [74–76].

Mutations and constitutive activation of the MAP kinase pathway have been of interest of late. BRAF (in papillary thyroid cancer) and RAS genes in the MAP kinase pathway normally code for growth and function in normal and tumor cells. BRAF mutations have been identified in approximately 45% or more of clinically evident papillary carcinomas and may behave more aggressively [77–79]. Activating mutations of RAS are more common in follicular variant PTC and follicular thyroid cancer [80] and may be a marker of more aggressive disease [81].

Other discoveries include the dependence of tumors on angiogenesis. Angiogenesis is important for tumor cell growth, promotion, and development of metastases [82]. Vascular endothelial growth factor (VEGF), an important proangiogenic factor, binds to VEGF receptors that in turn can further activate MAP kinase signaling and promote further tumor growth. VEGF receptors play a contributory role in the development and progression of thyroid cancer [73, 83]. VEGF expression is associated with higher risk of recurrence and shorter disease-free survival [84, 85]. Like in other tumors, epigenetic modifications of chromosomal DNA and histones, including the promoter gene of the sodium-iodine symporter, may also play an important role in promotion of tumor growth.

**(2) Therapeutic Options and Clinical Trials**

Patients with progressive or symptomatic metastatic thyroid cancer that is deemed nonradioiodine responsive should be considered for treatment on a clinical trial [5]. The recent identification of the molecular and cellular pathogenesis of both the development and progression of cancer has led to development of newer molecular-targeted therapies. Oncogenic mutations in the MAP kinase pathway (BRAF and RAS), as well as the importance of vascular endothelial growth factor receptors in thyroid cancer (mentioned above), have led to several clinical trials with small molecule inhibitors. These agents inhibit multiple kinases and can affect multiple signaling pathways. Tyrosine kinase inhibitors (TKIs) are orally administered and generally well tolerated. Immunomodulators, other oncogene inhibitors, and modulators of growth or apoptosis are all under investigation as well. Various clinical trials in the United States and Europe are recruiting differentiated thyroid cancer patients with radioiodine-negative progressive disease. The following are among the ones that have raised maximal interest and are summarized in Table 3.

**(3) Commercially Available TKIs**

(a) *Sorafenib*. Sorafenib is an oral, small molecule tyrosine kinase inhibitor that inhibits RET, BRAF, and VEGF receptors 2 and 3. It is currently approved in the United States for advanced renal cell carcinoma and unresectable hepatocellular carcinoma. Two phase II trials have been performed in patients with differentiated thyroid cancer that have both shown promise. In the larger, National Cancer Institute sponsored study with 58 patients with differentiated and anaplastic thyroid cancer (46 differentiated thyroid cancer patients evaluable for response). The partial response rate was 13%, and stable disease rate was 54% in patients with differentiated thyroid cancer; however, 6 patients who were deemed not assessable were excluded from this analysis [86]. A second phase II study reported partial remission in 7 of 22 (32%) patients with differentiated thyroid cancer [87]. With early data showing sorafenib to have promise in differentiated thyroid carcinoma (DTC) and sorafenib being a commercially available drug, many clinicians began using this drug in an off-label manner (not FDA approved for thyroid cancer). We recently reviewed our experience with off-label use of sorafenib in patients with DTC [88]. All patients had progressive nonradioactive iodine-avid disease to receive drug. Twenty percent of the patients developed a partial response, and 60% of patients developed stable disease achieving a clinical benefit rate of 80%. Progression-free survival was lengthened from four months predrug to 19 months. There was no difference in response based on BRAF mutational status in any of these studies thus far. A phase III international randomized controlled trial is underway currently evaluating sorafenib in progressive nonradioiodine-responsive metastatic differentiated thyroid cancer. This trial is randomized to placebo, and the primary endpoint is progression-free survival.

(b) *Sunitinib*. Sunitinib is another oral small molecule tyrosine kinase inhibitor that is FDA approved for the treatment of metastatic renal cell carcinoma. This drug inhibits RET, RET/PTC subtypes 1 and 3, and VEGFR [89]. Two patients with differentiated thyroid carcinoma treated with daily sunitinib for four weeks and two weeks holiday have had prolonged partial responses and decreases in SUV on PET scan [90]. An ongoing open-label phase II study showed a 13% partial response rate, and in another 68% percent of differentiated thyroid carcinoma patients, disease stabilization was seen [91]. A second phase II study has reported partial remission or disease stabilization in two of 12 patients thus far [92]. Other case reports have described prolonged partial responses [90]. The study by Carr et al. is the largest published trial to date using sunitinib in patients with metastatic PET-positive, RAI-refractory differentiated thyroid cancer. 28% of patients with differentiated thyroid cancer achieved a response (complete or partial response) [93].

(c) *Pazopanib*. Pazopanib is a small molecule inhibitor of all VEGFR subtypes and PDGFR. It is approved in the United States for the treatment of advanced renal cell carcinoma. Mayo Clinic recently reported on 37 rapidly progressive DTC patients on a phase II single agent trial [94]. 49% of patients had partial responses with a starting dose of 800 mg. Progression-free survival was 12 months. This drug carries a black box warning due to severe and fatal hepatotoxicity observed in a renal cell carcinoma patient. Liver transaminase levels should be monitored closely with this drug.

(d) *Adverse Events Common to Sorafenib, Sunitinib, and Pazopanib*. Adverse events seen with these drugs are similar to those described with the treatment of advanced renal cell carcinoma and are summarized in Table 4. The most common ones include diarrhea, hand-foot syndrome, rash, hypertension, and fatigue. Skin changes have also been noted including keratoacanthomas and squamous cell carcinomas in about 5–11% of patients taking sorafenib. Due to the promise shown by these agents in differentiated thyroid carcinoma and the availability and tolerability of these drugs, sorafenib, sunitinib, and pazopanib have been added as treatment options for patients with progressive nonradioactive iodine-avid thyroid cancer who cannot be enrolled in a clinical trial [5]. Due to significant potential toxicity, only clinicians versed in the management of the side effects of these therapies should use these drugs in a permissible off-label manner.

**(4) Clinical Trials with TKIs**

(a) *Motesanib*. Motesanib is an oral tyrosine kinase inhibitor that inhibits VEGFRs 1, 2, and 3 [95]. It is currently not commercially available. A phase II trial was initiated based on responses in five patients with differentiated thyroid cancer in a phase I trial [96]. Patients with progressive differentiated thyroid cancer based on serial radiographic imaging in a six-month period were enrolled in this international phase II trial. 93 patients were enrolled, 14% had a partial response, and 35% of patients developed disease stabilization for 24 weeks [97]. One-third of the patients were still on drug after 48 weeks. The median progression-free survival was 40 weeks. Tumors with BRAF mutations responded better than those without, despite the lack of BRAF inhibition by motesanib, suggesting that these BRAF-positive tumors may be more dependent on VEGF-mediated angiogenesis. The most common adverse events noted include fatigue, nausea, diarrhea, and hypertension. This trial also noted the unanticipated side effect of a 30% increase in levothyroxine requirement to maintain the purposeful TSH suppression; two-thirds of the patients developed a TSH outside of the range.

(b) *Axitinib*. Axitinib is an oral tyrosine kinase inhibitor that blocks VEGFRs and has been studied in thyroid cancer. It is not commercially available. A phase II, multicenter study was initiated based on the experience of five thyroid cancer patients in a phase I trial [98]. 31% of differentiated thyroid carcinoma patients had a partial response [99]. Many of the patients had been previously treated with various chemotherapeutic agents. Median progression-free survival for all histologic types of thyroid cancer was 18 months. The most common side effects seen in this trial include hypertension, stomatitis, fatigue, and diarrhea. A current trial is underway to evaluate axitinib's efficacy in doxorubicin-refractory metastatic thyroid cancer patients.

(c) *Other Therapeutic Agents*. A small molecule inhibitor of the epidermal growth factor receptor, gefitinib, has been looked at in advanced thyroid cancer but had no complete or partial responses [100]. With increased expression of c-MET described in PTC, cabozantinib (XL184), an oral small molecule inhibitor of various tyrosine kinases including C-MET and VEGFR2, is being studied in differentiated thyroid cancer [101–104]. Results presented at the American Thyroid Association (ATA) were very encouraging, with a partial response rate of 53% in a cohort of 15 DTC patients [101]. A phase 2 trial with lenvatinib (E7080) in 58 patients with radioactive iodine refractory DTC showed 50% partial response and a progression-free survival of 13 months [105]. XL281, a small molecule inhibitor of BRAF kinases currently in a phase I trial, preliminarily shows five patients with papillary thyroid carcinomas with prolonged stable diseases. Two of these patients had V600E BRAF mutation [106]. Vemurafenib (also known as PLX4032, RO5185426, and RG7204), also a small molecule inhibitor of mutant BRAF kinase, has shown promise in thyroid cancer [107] and is currently being studied in patients with BRAF-mutated thyroid cancer in a phase II trial. An open-label phase II study in Kentucky examined the efficacy of thalidomide in progressive metastatic thyroid cancer of all histologies [108]. An 18% partial response rate with 32% stable disease as best response is described in the 28 evaluable patients of all thyroid histologies. Lenalidomide, a similar drug to thalidomide but less toxic, is being evaluated in a phase II open-label study in DTC patients currently [109]. Thus far, 39% of the patients developed a partial response and 50% in whom disease stabilized. Overall survival was shorter than in most trials with a median of 11 months.

(d) *Agents to Restore Radioactive Iodine Uptake*. Researchers have been searching for ways to restore loss of radioactive iodine to nonavid tumors. 13 cis-retinoic acid partially restored radioactive iodine uptake in poorly differentiated follicular thyroid cancer cells [110]. Many clinical trials using retinoid receptors and other drugs have been focused on this restoration of iodine avidity with little success. Bexarotene, a synthetic agonist of the retinoid X receptor, was evaluated in a phase II trial to attempt to restore radioiodine activity, followed by treatment. After six weeks of therapy, eight of 11 patients had partial restoration of iodine avidity, but there was not much tumor reduction [111]. Rosiglitazone, a peroxisomal proliferators-activated receptor-gamma agonist, was evaluated for restoration of radioiodine uptake [112]. After eight weeks of treatment, although four patients had iodine avidity, clinical response was lacking. Depsipeptide, a histone deacetylase inhibitor, was evaluated in a phase II trial of patients with nonradioiodine metastatic DTC [113]. Only one patient out of 14 showed improvement in radioiodine uptake, but significant cardiac toxicities were seen, including sudden death. The most encouraging of these studies was presented at the American Thyroid Association (ATA) in 2011, evaluating single-agent MEK1/2 inhibitor, selumetinib (AZD6244), in 17 patients who were RAI refractory. In 11 patients, RAI uptake was restored. Information on best response was available in 7 patients, 6 of whom had a partial response to RAI [114].

Other agents are under investigation in both phases I and II studies, including agents that inhibit the PI3kinase/aKt pathways, histone deacetylase inhibitors, and combinations of methylation inhibitors with histone deacetylase inhibitors.

## 4. Summary

Differentiated thyroid carcinoma that is nonradioiodine avid is difficult to detect and treat. Clinically dictated selected imaging including ultrasound of the neck, CT imaging of the chest, MRIs of the spine and brain, bone scan, and ^18^FDG-PET-CT have all been useful in the detection of disease. Further radioactive iodine therapy in these patients tends to increase adverse events with minimal clinical benefit. 

Recurrent neck disease is often treated with further surgery. In addition, patients may benefit from resection of specific symptomatic metastatic sites. Selected patients may benefit from external beam radiotherapy, radiofrequency ablation, or chemoembolization of other metastatic sites as well.

Patients with stable metastatic disease may be observed with thyroid hormone suppression therapy only. More advanced, progressive neck disease and progressive, distant metastatic disease require systemic treatment. Cytotoxic chemotherapy has limited response rates and significant toxicity and is therefore reserved for symptomatic progressive disease in a patient that cannot get on a clinical trial or tolerate antiangiogenic therapy. The advancement in understanding the molecular aberrations in thyroid cancer has led to an explosion of promising recent clinical trials. Targeted agents against the VEGF receptor and the MAP kinase pathway are amongst the most promising thus far. These agents have shown some of the most impressive responses and have fairly tolerable adverse effects. 

Given our current knowledge and trial results, it remains difficult to choose the optimal therapy for selected patients. Many of the trials had varying entry criteria, many of which did not require progression. No trial thus far has overall survival as the primary endpoint, prolongation of this being the ultimate goal of patients. In addition, most patients eventually progress through these agents suggesting development of other pathways of resistance. Future trials will likely necessitate combination of therapy with minimal increased toxicity. Future trials may include agents that inhibit the PI3 kinase pathway in addition to the MAP kinase pathway or combination of cytotoxic chemotherapy with targeted agents. The main goal of all these trials should be to prolong life with minimal decrease in quality of life.

## Figures and Tables

**Table 1:** Imaging modalities for RAI-refractory recurrent disease.

Imaging study	Utility	Pros	Cons
Ultrasound neck	Detection of neck disease	Sensitive; ability to biopsy	Operator dependent; difficult to detect invasive disease and disease in the posterior neck
CT	Detection of local and metastatic disease	Sensitive; less operator dependent	Radiation exposure; risk of renal injury with contrast; delays in radioiodine administration
MRI	Detection of local and metastatic disease	Sensitive for CNS disease; no radiation exposure	Difficult to tolerate in some patients; risk of nephrogenic systemic fibrosis (NSF) in patients with renal failure; contraindicated in patients with certain metal devices or implants
FDG-PET scan	Detection of metastatic disease and providing prognostic information	Sensitive when used with CT	Detects FDG-avid disease only

**Table 2:** Therapeutic modalities for RAI-refractory recurrent disease.

	Indication	Pros	Cons
Surgery	Surgically resectable local recurrences; metastasectomy	Potential for cure	Potential significant morbidity
External beam radiation	Adjuvant: neck Therapeutic and palliative: metastatic sites	Decrease in recurrence, progression, and pain	May preclude future neck surgery; dysphagia and xerostomia; secondary malignancies
PEIT	Locally recurrent disease in patients at high risk for morbidity and mortality from surgical resection	Potential for avoidance of surgery	Local pain; injury to local structures; unknown effect on survival and recurrence
Systemic chemotherapy (including TKIs)	Unresectable, RAI-refractory, metastatic disease	May slow progression of disease; may alleviate disease symptoms	Significant adverse events; unknown effect on survival

PEIT: percutaneous ethanol injection therapy; TKI: tyrosine kinase inhibitors.

**Table 3:** Targeted therapies evaluated in clinical trials for thyroid cancer.

Drug	VEGFR1	VEGFR2	VEGFR3	RET	BRAF	Other	Response; PFS	Citation
Axitinib	X	X	X				31% PR; 18 mos (MTC included)	Cohen et al. [91, 99]

Motesanib	X	X	X	X			14% PR; 9 mos	Sherman et al. [97]

Sorafenib		X	X	X	X		13–32% PR; PFS 10–21 mos	Kloos et al. [86], Gupta-Abramson et al. [87], Cabanillas et al. [88]

Sunitinib	X	X	X	X			28% CR + PR; TTP 13 mos	Carr et al. [93]

Pazopanib	X	X	X	X			49% PR; PFS 12 mos	Bible et al. [94]

Lenvatinib	X	X	X	X		FGFR	50% PR; PFS 13 mos	Sherman et al. [105]

Cabozantinib	X	X		X		c-MET	53% PR; PFS n/a	Cabanillas et al. [101]

PR: partial response, SD: stable disease, TTP: time to progression, PFS: progression-free surivival, n/a: not available, and mos: months.

**Table 4:** Adverse events associated with the commercially available TKIs used in thyroid cancer.

Adverse event	Sorafenib (%)		Sunitinib (%)		Pazopanib (%)	
	All grade	≥grade 3	All grade	≥grade 3	All grade	≥grade 3
Hypertension	17	4	30	12	40	4
CHF or LVEF decline	1.7	NR	13	3	<1%	NR
Proteinuria	NR	NR	NR	NR	9	<1
Hand-foot skin reaction	30	6	29	6	6	NR
Stomatitis	NR	NR	30	1	4	NR
Anorexia	16	<1	34	2	22	2
Weight loss	10	<1	12	<1	52	3.5
Diarrhea	43	2	61	9	52	3.5
AST elevation	NR	NR	56	2	53	7.5
ALT elevation	NR	NR	51	2.5	53	12
Fatigue	37	5	54	11	19	2
Hypothyroidism	NR	NR	14	2	7	NR
Arterial thromboembolism	2.9	NR	NR	NR	3	2
Hemorrhage/bleeding (all sites)	15	3	30	3	13	2

CHF: congestive heart failure; LVEF: left ventricular ejection fraction; AST: aspartate aminotransferase; ALT: alanine aminotransferase; NR: not reported. table adapted from [115].
